# Speed Overestimation of the Moving Away Object in the *Intentional Reaction* Causal Effect

**DOI:** 10.1177/2041669520980019

**Published:** 2020-12-26

**Authors:** Giulia Parovel, Stefano Guidi

**Affiliations:** Department of Social, Political and Cognitive Sciences, University of Siena, Siena, Italy

**Keywords:** causal perception, psychological causality, animacy, intentional reaction effect, speed perception, speed overestimation, chasing, escaping

## Abstract

We describe a new illusory speed effect arising in visual events developed by Michotte (1946/1963) in studies of causal perception and, more specifically, within the so-called *intentional reaction effect*: When an Object *B* is seen intentionally escaping from another Object *A*, its perceived speed is overestimated. In Experiment 1, we used two-alternative forced choice comparisons to estimate perceived speed scale values for a small square moving either alone or in different contexts known to elicit different impressions of animacy (Parovel et al., 2018). The results showed that *B*’s speed was overestimated only in the condition in which it moved away from another approaching square moving in a nonrigid way, like a caterpillar. In Experiment 2, we psychophysically measured the magnitude of speed overestimation in that condition and tested whether it could be affected by further animacy cues related to the escaping object (the actual velocity of the square) and to the approaching square (its type of motion: caterpillar or linear). Results confirmed that *B*’s speed was overestimated up to 10% and that the degree of overestimation was affected by both experimental factors, being greater at higher speeds and when the chasing object moved in an animate fashion. This speed bias might be related to a higher sensitivity of the visual processes to threat-related events such as fighting and chasing, leading to evolutionary adaptive behaviours such as speedy flight from predators, but also empathy and emotion understanding.

In the classical Michotte’s *launching effect*, when an Object A moves toward, and makes contact with, another Object B, which then moves away with a slower speed, B is perceived as it were pushed by A in a mechanical collision and its motion looks passive (Costall, 2014; Michotte, 1946/1963). When the speed of B exceeds the speed of A, the launching effect gives way to the *triggering effect*, which refers to the impression that the motion of B, even if somehow caused by A, is active and self-propelled (Michotte, 1946/1963). This stimuli configuration led to the discovery of a different form of causality that was not mechanical but psychological, because it is related to impressions of animacy and of intentional states. These impressions are most evident in the *intentional reaction effect* (Kanizsa & Vicario, 1968), where B’s motion starts before the contact with A, and B is seen as *intentionally escaping* from A, in an *action–reaction* event seen as psychological or social causality (Schlottmann & Ray, 2010; Schlottmann & Surian, 1999; Schlottmann et al., 2002, 2006, 2012, 2013).

In the light of a broad body of research, perception of causality and perception of animacy seem to be irresistible and dependent entirely on basic display parameters and to emerge even when individuals lack prior knowledge (e.g., in infancy; Choi & Scholl, 2006; Leslie, 1984; Leslie & Keeble, 1987; Mascalzoni et al., 2013; Schlottmann et al., 2012; Scholl & Nakayama, 2002; Scholl & Tremoulet, 2000; Wagemans et al., 2006). As Scholl and Gao (2013) argued, such phenomena of causality and animacy reflect truly visual processing as opposed to higher level judgement and categorization based on visual input (see also Hubbard, 2013a, 2013b; Rolfs et al., 2013; Wagemans et al., 2006).

One of the crucial low-level parameters for the perception of causal events is spatiotemporal contiguity between the two moving objects, which seems to act as a factor of grouping (Bae & Flombaum, 2011; Choi & Scholl, 2004; van Buren et al., 2017). The gestalt psychologist Karl Duncker (1935/1969) had already observed that even two unrelated events can be grouped in a cause–effect relationship on the basis of their temporal coincidence alone, as sometimes it happens in daily life (e.g., when a gust of wind suddenly shuts the door and in the same instant a light comes on in the opposite side of the hallway). According to several authors, in these cases, causal grouping would depend on a coincidence avoidance principle that is a tendency of the visual system to avoid interpretations that involve coincidences regarding the proximal stimuli. This principle would have a central role in causal effects, like in causal capture (Scholl & Nakayama, 2002), but also in simple launching events, where the spatial contiguity and temporal contingency between the two objects and their movements is seen as nonaccidental (Choi & Scholl, 2006).

At the same time, not only causal events are strictly dependent on perceptual parameters, but it seems that causal perception can also have an influence on the perceived low-level spatiotemporal features of the scene. In spatial binding of collision events, for instance, two objects appear closer in space when they are causally connected (i.e., the subjective size of the rectangle separating cause and effect in space would shrink; Buehner & Humphreys, 2010). In launching events, instead, the degree of overlapping between the two items is underestimated, and Scholl and Nakayama (2004) found that the degree of underestimation is higher when the causal nature of the event is induced by a surrounding context. In launching events, moreover, there can also be a systematic error in remembering the vanishing position of the second moving object during its postcollision motion (De Sá Teixeira et al., 2008; Hubbard et al., 2001; Hubbard & Ruppel, 2002). About speed, if Michotte (1946/1963) demonstrated the crucial role of objective speed ratio between A and B in perception of causality (see also Schlottmann & Anderson, 1993), apparent kinematics itself seems to be biased in causal events: Several works suggested that the perceived speed of B can be influenced by the speed of A, coherently to the naive physics of collisions and impetus theories (De Sá Teixeira et al., 2008; Hubbard, 2013a, 2013b; Parovel & Casco, 2006; see also Vicovaro, 2012, 2018; Vicovaro et al., 2020; Vicovaro & Burigana, 2014, 2016) and to White’s (2009) property transmission hypothesis. Parovel and Casco (2006) showed that the speed of the second object in causal events was overestimated for a wide range of speeds of the first object (launcher) but accurately assessed in noncausal events.

The goal of our work is to show a new speed overestimation effect in some types of causal events, and more specifically in the intentional reaction effect, an action-and-reaction sequence in which B’s motion starts before the contact with A, and B is seen as suddenly intentionally escaping from A (Kanizsa & Vicario, 1968; Parovel & Guidi, 2015; Schlottmann & Ray, 2010; Schlottmann & Surian, 1999; Schlottmann et al., 2002, 2006, 2012, 2013). In the intentional reaction, similarly to the trigger effect (Michotte, 1946/1963), the square B is faster than A, but the two squares do not come in contact with each other; moreover, the square B looks vividly endowed with animacy and psychological traits, similarly to Heider and Simmel’s (1944) classical demonstration.

We previously found (Parovel et al., 2018) that the impression of *animacy* in dynamic configurations (i.e., the impression of aliveness of a moving object) is strongly related to the spatiotemporal contingency between a target event and a context event. Also, Tremoulet and Feldman (2006) have shown, in different experimental displays, that animacy attributions can be either elevated or suppressed by the nature of the environment and the target’s interaction with it. In our work (Parovel et al., 2018), if a small square (B) moves on the screen alone or in the context of another square (A), which is either static or moving in an *animate,* or a *mechanical* way, the attribution of animacy to B differs: Animacy is more likely to be attributed when the motion of the target is causally related to the motion of the contextual object, just like the motion of B after the contact with A appears mechanically caused by A in the Michotte’s launching effect. Interestingly, in the same research, many participants’ spontaneous reports referred to different speeds of the moving squares in the different context conditions, suggesting that some squares “looked more animate because they moved more quickly,” while the actual speed of the square B *was always constant*. Coherently with these descriptions, many studies have shown an association between speed and animacy in that objects moving faster (Szego & Rutherford, 2007, 2008), or changing their speed (Träuble et al., 2014), are judged as more animate. In our study, we also found a significant effect of the direction of the target’s motion with respect to the context in that an escaping moving object looked more animate than an approaching one (Parovel et al., 2018). We hypothesized that the moving away behaviour may look more animate for evolutionary reasons because of a higher sensitivity to aggressive or threat-related events such as fighting and chasing, or for a general predisposition to note emotionally negative events, as the *negativity bias* theories predict (Heberlein, 2008). Since escaping predators is crucial for survival, it is plausible that evolution might have selected mechanisms for the acquisition of appropriate responses to environmental threats from visual observation of such events (i.e., an object escaping from another chasing object).

Starting from these results, we aimed to see if—in the same experimental conditions—an escaping moving object, in our stimuli a square, could look not only more animate but also faster than an approaching one, that is, if there can be a specific association between speed and animacy in the moving away condition in which the second moving object starts to move at the arrival of the first one. To test these hypotheses, we designed and conducted the two experiments reported in this article. In the first one, we wanted to verify empirically that within the set of stimuli used in our previous study (Parovel et al., 2018), the same conditions in which a geometric shape is seen as more animate are the ones in which the shape is perceived as moving faster, despite its actual speed being constant across the different conditions. The second experiment was then conducted to come to a first estimate of the magnitude of the illusory speed difference and test the relationships between this effect and some factors known to affect perceived animacy.

## Experiment 1

The goal of Experiment 1 was to test, using two-alternative forced choice comparisons, whether and how the perceived speed of a target square varies across a set of stimulus conditions in which, according to our previous research (Parovel et al., 2018), the square is perceived more or less animate but has the same physical speed.

### Methods

#### Participants

Twenty-three students at the University of Siena participated in the experiment on a voluntary basis. All participants reported normal or corrected-to-normal vision. All gave their written informed consent in accordance with the Declaration of Helsinki and were debriefed at the end of the experiment about the purpose of the study. The Internal Review Board of the University of Siena reviewed the experimental protocols and approved the study.

#### Design and Stimuli

The stimuli used in the experiment were the same eight brief animations used in Parovel et al. (2018) realized in Adobe Flash Professional CS 5.5 and presented as QuicktimeTM displays, in which a small black square (henceforth also referred to as the target square) moves horizontally within a white rectangular space, either alone (*no context*) or in the context of a second grey square (henceforth also referred to as the context square), which, in turn, is either static (*static context*) or moves in different ways, vertically from top to bottom (simulating a fall: *mechanical context*) or horizontally in an animate fashion (contracting and expanding while moving, like a caterpillar: *animate context*). In fact, nonrigid, by rhythmic expansion–contraction motion, has been shown able to evoke a vivid organic motion, similarly to a caterpillar (e.g., Hubbard & Ruppel, 2002; Michotte, 1946/1963; Schlottmann & Ray, 2010). The context square moved towards the right while, at the same time, alternating phases of horizontal expansion and contraction. The size of the two squares on the screen was identical (side = 0.5°). The black and grey squares were placed inside a white rectangle (width = 12.2°, height = 8°) placed at the centre of the screen against a grey background. At the onset of the stimuli, the black square was placed on the horizontal midline of the rectangle, at a distance from the left border varying between 0.5° and 4.5°, according to the experimental condition. The motion of the black square was smooth, and its speed was constant (3.37°/s), and it stopped after a displacement of 4.5°. The speed of the context square was 3°/s. In the dynamic contexts, the motion of the black square started 500 ms after the context square started to move. In the no context and in the static context condition, the black square started to move after 660 ms from the onset of the stimulus.

**Figure 1.**
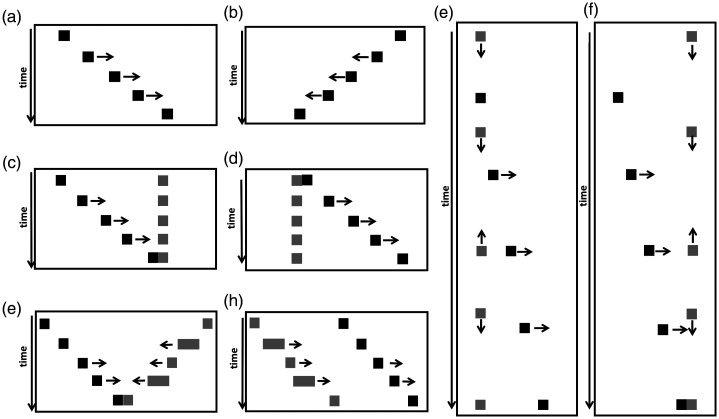
Schematic depictions of the stimuli used in Experiment 1. In each panel are represented the relative positions and directions of motion of the black square (target) and of the grey square (context) at different consecutive time-points during an animation. In (*a*) and (*b*), there is no context element. In (*c*) and (*d*), the context square is static. In (*g*) and (*h*) and in (*e*) and (*f*), the context is dynamic, and the context moves respectively in an animate fashion (contracting and expanding while moving like a caterpillar, *animate context*) and falling down from above and bouncing (*mechanical context*). In (c, g, and e), the black square (target) moves towards the context square, while in (d, h, and f), it moves away from it. The full set of displays is available online as Supplementary Materials: EXP1-a, EXP1-b, EXP1-c, EXP1-d, EXP1-e, EXP1-f, EXP1-g, and EXP1-h.

A schematic representation of the stimuli is presented in Figure 1. In the three conditions in which the context square is present, the black square moves either towards the context square (*c*, *g*, and *e*) or away from it (*d*, *h*, and *f*), while in the other condition, it moves either from left to right or from right to left. Overall, the eight stimuli correspond to all the possible combinations of two factors: context (four levels) and direction of motion of the black square with respect to the context square (two levels: moving towards or moving away from it).

The experiment consisted of 56 trials, divided into two 2 blocks, each one comprising all the 28 possible pairwise combinations of the 8 stimuli (8(8–1)/2 = 28). The order of the stimuli in the pairs was balanced across blocks, and the order of trials in each block was randomized. The stimuli were presented on a LED ultrabook monitor, with a screen measuring 13.3 inches diagonally, and participants viewed them from approximately 60 cm, with unrestricted head and eye movements.

#### Procedure

At the beginning, participants of the experiment were told that on each trial they would see two simple animations, one after the other, and that their task would be to choose the one in which the black square appeared to move more quickly. At the beginning of each trial, the screen remained blank for 500 ms, and then the first display was played. After the first display stopped, the squares were removed, and the white rectangle remained empty on the screen for another 1,500 ms, before the second display was played. After the second movie stopped, the screen turned blank again, and instructions were shown to the participants: They were told to indicate the movie in which the black square had seemed to them to move more quickly, by pressing the left key if they thought it was moving faster in the first movie and the right key if they thought it moved faster in the second movie. After completing a block of trials, participants were given the opportunity to take a break if they desired. Stimuli presentation and data collection during the experiment were controlled by PsychoPy2 v1.80.01 software running on an Acer Aspire S3 (Peirce, 2007; Peirce et al., 2019).

#### Statistical Analyses

Paired comparisons data were analysed using the Bradley–Terry–Luce (BTL) method (Bradley & Terry, 1952; Luce, 1959) and the elimination-by-aspects (EBA) method (Tversky, 1972) to derive ratio-scale values for perceived speed of the black square in the different conditions, with confidence intervals. These methods are extensions of the classical method of paired comparison first proposed by Leon Thurstone (1927) for the scaling of psychological qualities. Both methods try to model the probability of choosing one stimulus over another stimulus (as being, in our case, faster) in a pairwise comparison as a function of the location of both the stimuli on a psychological continuum (e.g., perceived speed) and, in the case of the EBA method, also of other perceived features of the stimuli. Goodness-of-fit tests are used to measure the success of the modelling attempts and to verify the possibility of ordering the stimuli on a ratio-level scale (with respect to the quality participants were asked to judge). Likelihood-ratio tests were used to assess the fit of the models to the data (by comparing the fitted model to the saturated model to check the assumption of the model concerning the consistency of participants’ judgements^1^) and test the presence of differences among stimuli in choice probability in the two-alternative forced choice trials concerning the speed of the black square (by comparing the fitted model to the model with equal probability of choice among all stimuli). The latter test can be considered akin to an *F* test in a one-way analysis of variance that when significant shows that at least one condition is different from another one in the measure of interest, while confidence intervals can be used to identify the conditions that are different. The EBA method is a generalization of the BTL method that allows to include extra parameters to account for (and explicitly model) possible similarities among the stimuli on one or more aspects (i.e., the fact that some stimuli share common features). These parameters are supposed to reflect how the difference (or similarity) between two stimuli in a trial concerning the presence of specific aspects can influence participants’ choice with respect to their apparent speed. To verify the presence of an effect of the order of presentation of the stimuli in a trial on participants’ judgements of perceived speed, we fit a generalized logistic mixed-effects model to the response data and estimated the odds of participants responding that the black square was moving faster in the second animation, with 95% confidence intervals. All the statistical analyses were performed using the software R version 3.5.1 (R Core Team, 2018) and the functions in the *eba* package (Wickelmaier & Schmid, 2004).

### Results and Discussion

We initially investigated the consistency of the participants’ judgements counting the number of Weak Stochastic Transitivity (WST) violations (see Note 1). No violations were found, and thus, it should have been possible to order the stimuli with respect to the perceived speed of the black square, and the attempt to estimate perceived speed scale values using probabilistic choice models was justified. The BTL model did not fit well to the data, χ^2^(21) = 40.5; *p* = .006. The EBA model including two extra parameters corresponding to (a) presence of a context (vs. no context) and (b) presence of a dynamic context (vs. a static context) instead had a good fit, χ^2^(19) = 26.15; *p* = .126, and the results showed that indeed in some conditions the choice probability (of the black square being considered faster than the one in the comparison stimulus) was different than in others. The extra parameters in the EBA model were only marginally significant, but the fact that the model had significantly higher fit than the BTL model showed that both the presence of a context (as opposed to no context) and the dynamicity of the context could have an effect on the probability of choosing a stimulus over another one (and thus on the resulting ratio-scale measures of the stimuli along the specified criterion, in our case the perceived speed), in the trials in which the stimuli differed with respect to these features. Finally, we tested the presence of a bias in the responses due to the order of presentation of the stimuli in the trials. The odds of participants responding that the black square was moving faster in the second animation estimated from fitting a generalized linear mixed-effects models (GLMM) model was 0.99 (95% CI [0.88, 1.1]), indicating that there was no bias in participants’ judgements due to presentation order.

**Figure 2.**
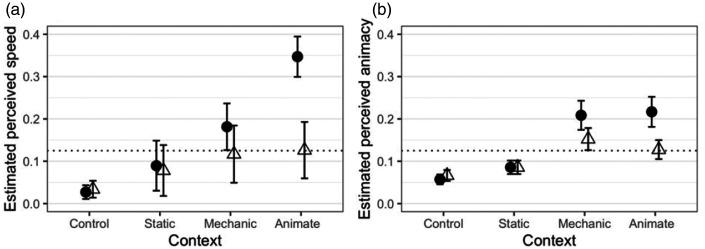
(a) Perceived speed scale values for the different conditions in Experiment 1, derived from the EBA model fitted to the paired comparison data. (b) Perceived animacy scale values for the same conditions, estimated by Parovel et al. (2018). In both plots, the error bars represent 95% confidence intervals for the estimated values. The dotted lines indicate the location on the scale where all the stimuli would lie if, for each pair of stimuli, the probability of choosing one over the other as faster/more animate was 0.5 (i.e., if there were no difference in the perceived speed or animacy of the black square across the conditions).

The scaled values of the perceived speed are plotted in Figure 2A, with 95% confidence intervals. As it can be seen in the plot, the perceived speed estimated from the comparison data was highest in the chasing condition (animate context, black square moving away), and clearly higher than in the other conditions. It was also somewhat higher in the mechanical context when the black square moved away. This suggests that some of the stimulus conditions that we tested are able to induce an overestimation of the speed of a moving object.

It is interesting to compare the scaled perceived speed values with the scaled animacy values estimated by Parovel et al. (2018) for the same stimuli that are plotted in Figure 2B. As it can be seen in the plot, the pattern of the perceived animacy values across the conditions is similar in several aspects to the pattern of the perceived speed values: The conditions in which the black square was seen as significantly least animate (the control and the static context conditions) are the conditions in which the black square was also perceived as slowest. Conversely, the highest perceived speed was found in the animate context conditions. Moreover, just as the perceived animacy in the dynamic context conditions in our previous study was higher when the black square moved *away* from the context square than what it moved *towards* it, also the perceived speed in these conditions tended to be higher with away-directed motion than with approaching motion. However, only in the animate context, when the contextual object was an animate object, this difference in the perceived speed across motion directions was statistically significant. Moreover, while the impressions of animacy of the black square moving away from the context element did not differ across the different types of dynamic contexts, the perceived speed did, and it was clearly and consistently higher in the animate context than in the mechanical context.

Overall, the current results, in conjunction with the findings of Parovel et al. (2018), seem to confirm the existence of a relationship between the perception of animacy of a visual shape and the perception of its speed that goes beyond what was previously hypothesized about stimulus velocity as being a factor contributing to whether an object is seen as animate or not (and to the strength of the animacy impressions). In our stimuli, in fact, the physical speed of the black square was always the same, and the types of contextual elements introduced in the experimental conditions were not factors that are known to be able to influence speed perception. Nonetheless, in conditions previously shown to produce the greater impression of animacy, an overestimation of speed was observed. Therefore, it seems that not only speed differences can trigger different impressions of animacy (Szego & Rutherford, 2007) but also that, vice versa, in specific stimulus conditions, different impressions of animacy can induce different estimations of speed. However, as noted earlier, the speed overestimation was only found in the escaping condition, and it was much stronger when the contextual element exhibited animate motion features. This seems to suggest that the perception of speed and the impression of animacy can be dissociated (see also Szego & Rutherford, 2008) and that speed overestimation is specifically linked to social interactions such as chasing.

More generally, the moving away speed bias could be related to a higher sensitivity of the visual processes to events such as chasing and escaping, or in general to events with an emotionally negative content (Heberlein, 2008; Walk, 1984). In addition, through the observation of survival-related behaviours such as speedy predator avoidance, this sensitivity may improve affective empathy and supporting social cognition. Threat-related events, such as fighting and chasing, according to Heberlein (2008), are related to the amygdala and may prompt a social interpretation of the Heider and Simmel (1944) movie. Moreover, in our previous work (Parovel et al., 2018), the analysis of free reports of the same displays adopted in our experiment, obtained by applying thematic coding to each description given by participants, showed that participants tended to attribute a negative emotion to the black square when it was moving away from the context and a positive one when it was approaching it.

The procedure used in the experiment, however, did not allow us to assess the degree of overestimation of the black square’s speed. We therefore designed and conducted a second experiment to measure the perceived speed of the black square with psychophysical methods.

## Experiment 2

The goal of Experiment 2 was to psychophysically measure the perceived speed of the black square in the condition of Experiment 1 (condition *h* in Figure 1) in which the speed overestimation was greater. In this condition, according to our previous study, the black square also appears animate (Parovel et al. 2018), looking like it were intentionally escaping from another approaching square, as it happens in *intentional reaction* or *social causality*, where a two-dimensional square moves towards another square, which gets away before the first square reaches it (Kanizsa & Vicario, 1968; see also Schlottmann & Surian, 1999). In adults and children from 3 years of age, this impression is amplified when the square moves like an animal, that is through nonrigid rather than rigid motion, as in Michotte’s caterpillar stimulus (Schlottmann et al., 2002, 2006). To verify whether also the degree of overestimation could be modulated by the perceived animacy, we also decided to vary other stimulus conditions that could affect the perception of animacy for the black square: the actual velocity of the black square and the type of motion of the context square (caterpillar or linear).

### Methods

#### Participants

Sixteen subjects (9 females and 7 males), mainly students at the University of Siena, participated in the experiment on a voluntary basis. The mean age of participants was 35.4 years (*SD* = 20.9), and all reported normal or corrected-to-normal vision. Participants gave their written informed consent in accordance with the Declaration of Helsinki and were debriefed at the end of the experiment about the purpose of the study.

#### Experimental Design, Procedure, and Stimuli

**Figure 3.**
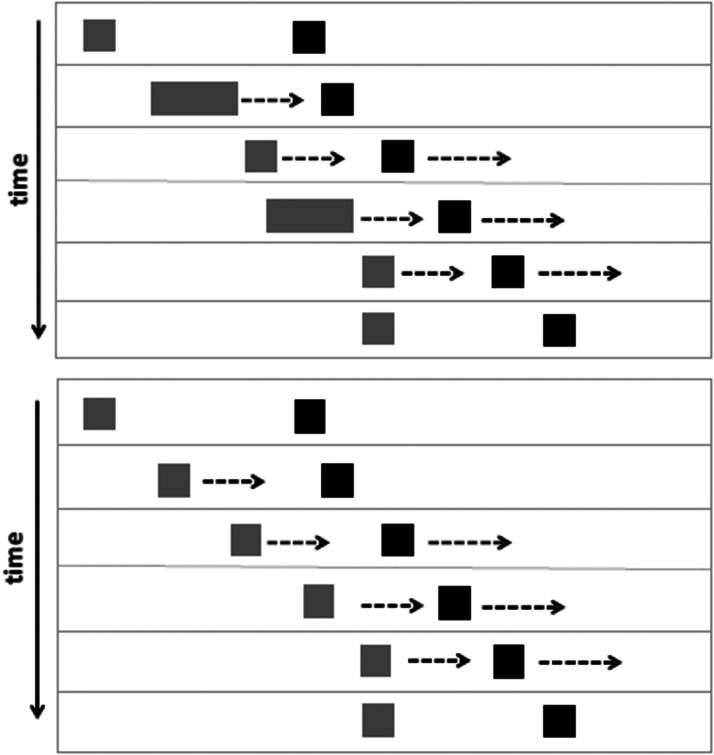
Frames of the two target animations, with caterpillar (top) and linear (bottom) motion of the grey square. The full set of target displays is available online as Supplementary Materials: Exp2-A, Exp2-B, Exp2-C, and Exp2-D.

We used the method of constant stimuli to measure the magnitude of the overestimation of the speed of a black square moving away from another approaching square in four conditions, derived from the factorial combination of the levels of two factors: the black square’s actual speed (*slow =* 3.37°/s and *fast =* 4.5°/s) and the type of motion of the approaching square (moving at about 3°/s speed either in a *linear* fashion or in a *caterpillar* way). In all the conditions (brief computer-generated animations realized in Adobe Flash Professional CS 5.5 and presented as QuicktimeTM displays), a small grey square (side = 0.5°), placed on the horizontal midline of a white rectangular space (the same of Experiment 1) at 0.5° from its left border, moved towards a black square (side = 0.5°), placed at 4.5° from the left border of the rectangle, along a straight path for 6.2°; when the distance between the two squares was 1.2°, the black square started to move towards the right for 4.5°. According to the free reports collected from the participants in the experiment conducted by Parovel et al. (2018), this spatiotemporal configuration produces the clear impression that the black square is moving away, escaping, from the grey object. This phenomenon is similar to the intentional reaction effect (Kanizsa & Vicario, 1968) and to other action-and-reaction sequences, situations described as psychological causation (Schlottmann et al., 2002, 2006). A schematic depiction of the stimuli in the linear and caterpillar conditions is presented in Figure 3.

For each experimental condition, 70 two-interval forced-choice trials were run, for a total of 280 judgement trials per participant. In each trial, in one of the two intervals (randomly determined), we presented one of the four brief animations previously described (chasing pair), and in the other interval, a comparison stimulus was shown in which only the black square was present (probe) and moved in the same direction as in the chasing pair, and along the same path, at one of seven possible speed levels. Just as in Experiment 1, participants’ task was to indicate, by pressing one of two keys, in which of the two intervals the black square seemed to move more quickly. The speed levels of the black square in the comparison stimulus (i.e., of the probe) were derived from the speed of the black square in the chasing pair. Starting from the actual speed level, we progressively increased/decreased the speed level by 15% increments/decrements (with respect to the previous level). For the slow condition, the levels were 2.07°/s, 2.44°/s, 2.87°/s, 3.37°/s, 3.87°/s, 4.45°/s, and 5.10°/s. For the fast condition the levels were 2.76°/s, 3.25°/s, 3.82°/s, 4.50°/s, 5.17°/s, 5.95°/s, and 6.85°/s. Each two-interval forced-choice trial (i.e., every combination of probe speed and chaser motion) was repeated 10 times. The trials were organized in 4 blocks comprising 70 trials each. The order of trials in the experiment was completely randomized by the PsychoPy3 (Peirce, 2007; Peirce et al., 2019) software that controlled the presentation of the stimuli and the collection of the responses, running on an HP Pavilion dm1-4125ea laptop. Participants viewed the stimuli from approximately 60 cm, with unrestricted head and eye movements.

#### Statistical Analysis

Paired comparison data were analysed using GLMM with a probit link function (i.e., the inverse function of the cumulative Gaussian distribution), following the approach described by Moscatelli et al. (2012). This method allows us to estimate the parameters of the psychometric function at the population level (instead than at the individual level) and compare them across experimental conditions in a single analysis, avoiding the need for a two-level approach (i.e., first estimating the parameters for each participant in each condition and comparing them across conditions with other statistical techniques such as repeated-measures analysis of variance).

The data from the slow and fast conditions were analysed separately, and in each analysis, we fitted a model including four fixed effects (respectively for the intercept, for the probe’s speed, for the chaser’s motion type, and for their interaction) and two random effects (respectively for the intercept and the slope for the probe speed), according to the following equation:

(1) $$\Phi^{-1}\left[P(Y_{ij}=\text{1}\right)]=\beta_{0}+u_{0i}+x_{ij}\left(\beta_{1}+u_{1i}\right)+d_{ij}\beta_{2}+\left(x_{ij}d_{ij}\right)\beta_{3}$$

where *Φ^−1^*[*P(Y_ij_ =* 1)] is the probit transform of the probability that Participant *i* in Trial *j* (when the probe’s actual speed was *x_ij_*) responded that the probe (i.e., the black square moving alone in the comparison stimulus) was faster than the black square in chasing pair stimulus. *d_ij_* is the dummy variable for the experimental condition, being 0 when the chaser’s motion was caterpillar (baseline) and 1 when it was linear, and *x_ij_d_ij_* is the interaction between the probe speed and the chaser’s motion. *β_0_* … *β_3_* are the fixed effects parameters, and *u_0i_* and *u_1i_* are the random effects parameters (i.e., the adjustments, respectively, to the intercept and the slope for Participant i). Further models with a different random structure (one including only a random intercept, and one including an additional random slope for the chaser’s motion slope) were also fitted to the data, but model comparison with likelihood-ratio tests showed that in both cases their fit to the data was worse than the model described in Equation 1. The point of subjective equality (PSE) and the just noticeable difference (JND) were derived from the parameters for the fixed effects estimated in the analyses, using the following equations:

(2) $$\mathrm{PSE}=\frac{\beta_{0}}{\beta_{1}}$$

(3) $$\mathrm{JND}=\frac{0.675}{\beta_{1}}$$

To compute confidence intervals for the PSE and the JND in the different conditions, we used the bootstrap method with 600 resamples.

PSEs in the different conditions were also estimated at the participant level, fitting a GLM model on the individual response data for each participant and using the formula in (2). The PSE estimates were then converted into the percent of overestimation of the speed of the black square in the chasing pair stimulus (with respect to its actual speed), subtracting the actual speed from the PSE values and dividing the result by the actual speed. These data were then analysed with linear mixed-effects models, including *target speed* and *chaser’s motion type* as factors, and a single random effect (relative to the intercept). More complex models with other random parameters were fitted, but likelihood-ratio tests showed that they had a worse fit than the simpler model.

All the statistical analyses were performed using the software R version 3.5.1 (R Core Team, 2018) and the functions in the *MixedPsy* package (Moscatelli & Balestrucci, 2017).

### Results and Discussion

**Figure 4.**
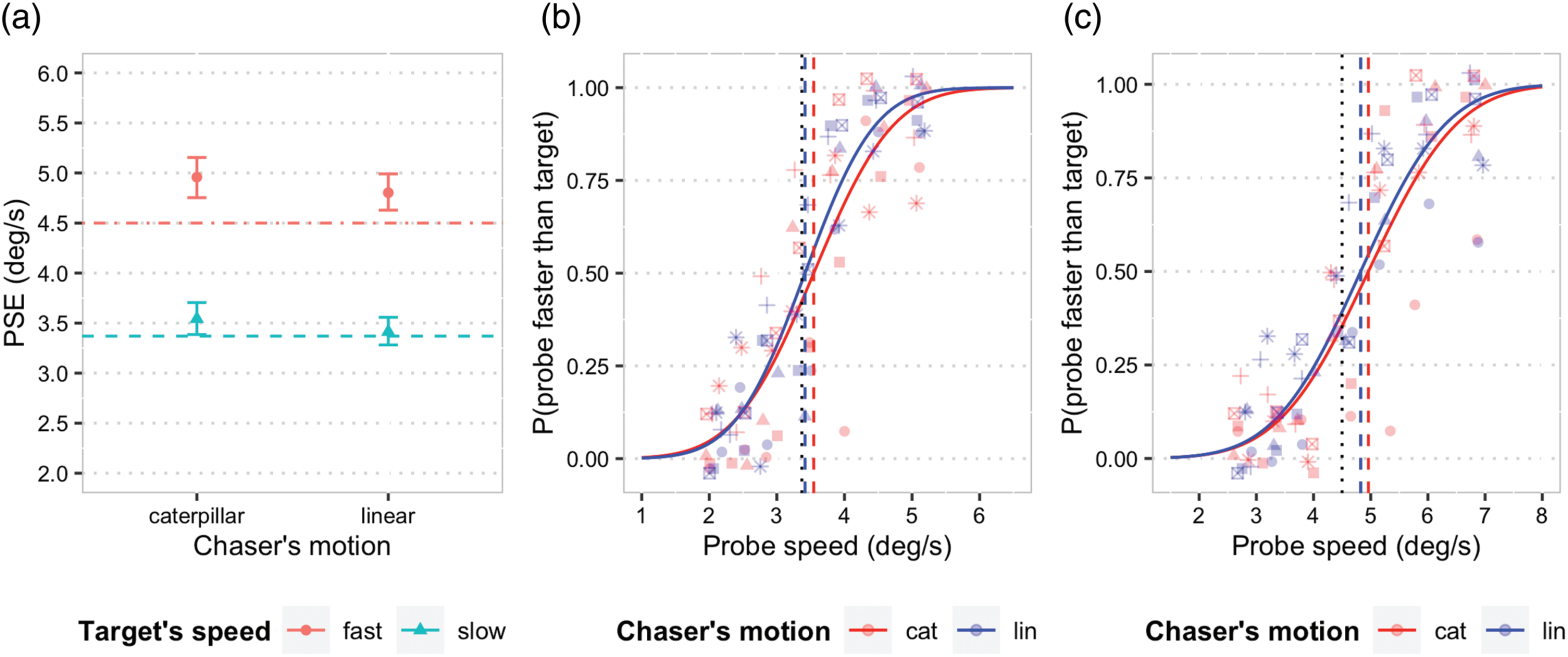
(a) PSEs estimates and 95% CI for the different experimental conditions; the dashed and dot-dashed horizontal lines represent the actual velocity of the black square (target) in the slow and in the fast condition, respectively. In (b) and (c) are plotted the psychometric functions fitted on the data from all participants, along with the individual data, respectively, in the slow and fast conditions. The dotted vertical lines represent the actual velocity of the black square in each condition, and the dashed blue and red vertical lines represent the estimated PSE for the linear and caterpillar motion, respectively. PSE = point of subjective equality.

Table 1 reports the results of the analysis of the data for both the slow and the fast conditions. For the fast condition, neither the chaser’s motion nor the interaction effect was significant. For the slow condition, instead, the results highlighted a marginally significant interaction effect (*p* < .1). For both types of chaser’s motion, the models had a good fit to the data. In Figure 4B and C are plotted the predicted response probabilities for all subjects, along with the actual response data, respectively, for the slow (4B) and fast (4C) conditions.

**Table 1.** Fixed Effects Parameters of the GLMMs Fitted on the Paired Comparison Data From Experiment 2, for Both the Slow and the Fast Condition.

Effect	Slow condition (AIC = 696.1)			Fast condition (AIC = 679.6)		
	*β*	*SD*	*p* value	*β*	*SD*	*p* value
Intercept (*β_0_*)	–3.97	0.32	<.001	–4.02	0.26	<.001
Probe speed (deg/s) (*β_1_*)	1.12	0.09	<.001	0.81	0.05	<.001
Chaser’s motion (= linear) (*β_2_*)	–0.31	0.28	.26	–0.08	0.28	.77
Probe Speed × Chaser’s Motion (*β_3_*)	0.14	0.08	.09	0.04	0.06	.45

*Note*. AIC = Akaike information criterion.

The PSE estimates for all the conditions are plotted in Figure 4A, along with 95% bootstrapped confidence intervals. As it can be seen in the figure for all the conditions, the estimated PSEs were numerically higher than the actual black square’s speed. In the fast condition, moreover, for both types of chaser motion, the 95% CI of the PSE did not include the actual speed (PSE_cat_ = 4.96; 95% CI [4.75, 5.16]; PSE_lin_ = 4.80; 95% CI [4.63, 4.99]), showing that in this condition the black square speed was overestimated between 6.7% (linear motion) and 10.2% (caterpillar motion). In the slow condition, however, the estimates of the PSE were much closer to the actual speed value. Moreover, only for the caterpillar motion, the 95% CI for the PSE did not include the actual speed of the black square (PSE_cat_ = 3.54; 95% CI [3.39, 3.71]), and thus the black square (target) speed was significantly overestimated, by 5.1%, while for the linear motion, the actual speed was inside the interval (PSE_lin_ = 3.41; 95% CI [3.28, 3.56]), and thus, the data in this condition did not provide evidence of an overestimation of the speed of the black square.

To better test the differences in the PSE between the experimental conditions, we conducted a further analysis. We first computed the PSEs estimates for each participant in each condition, by fitting a logistic model on the individual response data and extracting the model coefficients. We then computed from the PSE data the percent of speed overestimation with respect to the actual speed of the black square in each condition, to make possible comparisons between and across speed levels. Finally, we analysed the percent data (a measure of the degree of overestimation) with linear mixed-effects models, including both black square’s speed and chaser’s motion type as factors. The results showed significant main effects of both black square’s speed, *F*(1, 48)=22.03; *p* < .001, and chaser’s motion type, *F*(1, 48)=9.15; *p* < .01, and no significant interaction. In the fast condition, the percent of overestimation was 6 percent points higher (*SD* = 1.3) than in the slow condition. In the caterpillar motion condition, the percent of overestimation (8.2% across black square’s speeds) was 3.8 percent points higher (*SD* = 1.3) than in the linear motion condition (4.4% across black square’s speeds).

The results of Experiment 2 confirmed a tendency to overestimate the speed of an object (the black square, namely, the target) that moves away from another approaching object, a situation that our previous study (Parovel et al., 2018) showed viewers tended to interpret as chasing events in which the black square is seen as animate and escaping. Evidence for the overestimation was found in all the conditions but one. The experiment also allowed us to quantify the magnitude of this effect. The degree of overestimation was higher when the speed of the escaping square was higher. There could be different reasons for this effect. On one hand, it might be that the impression of chasing is much more evident when the black square’s speed is higher than the speed of the chaser. Alternatively, this effect might be the result of a greater perceived animacy of the escaping shape at higher speeds. It is in fact known that increase in speed is one of the factors that can influence, and increase, the impression of animacy of simple moving shapes. We also found that the overestimation was greater when the chasing object (i.e., the grey object) moved in an animate fashion (like a caterpillar) than when its motion did not convey this type of animacy cue. This was more evident, however, in the slow condition, when the PSE was significantly different, and higher, than the actual speed of the black square only if the chasing object moved as a caterpillar. It is thus possible that, in this condition, the speed of the black square was too low to induce the perception of escape in the absence of further contextual information, such as the animacy cue provided by the chaser’s pattern of movement. We can also hypothesize that the complexity itself of the caterpillar motion could play a role; further studies will be needed to test this hypothesis, although it may be very difficult to rule out the possibility that the effect of the caterpillar display may be due to some unknown low-level features rather than animacy per se.

Also other studies provided interesting explorations of the psychophysics of chasing (Gao et al., 2009, 2010; Gao & Scholl, 2011). They reported several experiments looking for the visual cues leading to the perception of chasing between two moving objects (the *wolf* and the *sheep*), or diminishing it, by adopting a different methodology based on dynamic visual search and interactive displays. Generally, this research indicates the nature and the limits of such perception and shows a dissociation between perceived chasing (i.e., the impression that an object is in pursuit of another moving object) and actual chasing (i.e., an object which consistently moves in the direction of another moving object—a form of objective pursuit). Their findings highlight the presence of strict spatial and temporal constraints on perceived chasing; chasing seems to be perceived efficiently whenever specific cues are at work (Gao et al., 2010) and not efficiently when those cues are not available. Gao et al.’s (2009) study, for instance, manipulated the angular deviation of the wolf’s heading relatively to the motion of the sheep (*chasing subtlety*), while Gao and Scholl’s (2011) results suggest that the degree of pursuit must be especially temporally cohesive and without disruptions to trigger the perception of chasing. It would be interesting to see if the speed overestimation effect here shown would apply even in a wider range of chasing displays and would be related to the constraints already found by these authors.

## Conclusions

The experiments described in this article investigated a new illusory speed effect in visual events derived from the ones developed by Michotte to explore causal perception, and more specifically in cases of psychological causality, such as intentional reaction (Kanizsa & Vicario, 1968). Kanizsa and Vicario’s (1968) reaction event embodies a simple action–reaction sequence: A two-dimensional square moves towards another square, which moves away before the first square reaches it, both moving simultaneously for about half a second (Kanizsa & Vicario, 1968). Observers see the first square chasing the second that is trying to run away, an impression amplified when the first shape moves through nonrigid motion by rhythmic expansion–contraction in a way that appears self-generated and animal-like (Schlottmann et al., 2002, 2006; see also Gao et al., 2009).

According to our results, moreover, an escaping moving object looks faster than an approaching or neutral one that is a single moving object in absence of any context; the second experiment allowed us to quantify the magnitude of the speed overestimation. The speed overestimation was found only in the escaping condition and not in the approaching one, and it was stronger when the contextual element, the chaser, moves like a caterpillar. Therefore, our results suggest that not only the impression of animacy but also other emotional attributions of the two movements, depending on the spatiotemporal structure of the event, can influence our perception of speed. We must however acknowledge that in the experiments described in this article, judgements about animacy were not collected. While we believe that the results of the experiment described in Parovel et al. (2018), which used the same stimuli used in our current Experiment 1 to explore the effect of different contexts on the perceptual saliency of animacy, provide evidence that in some conditions the target square (the black square) looks more animate (and particularly in the condition compatible with a chasing interpretation), further studies should try to replicate these findings by collecting both animacy and speed judgements from the same participants.

In the light of these results, we believe that speed overestimation effect could be added to the list of functional effects of causality on the low-level properties of the scene such as spatial binding (Buehner & Humphreys, 2010), avoiding overlapping (Scholl & Nakayama, 2004), and speed perception in the naive physics of collisions and impetus theories (Hubbard, 2013a, 2013b; Parovel & Casco, 2006; Vicovaro, 2012, 2018; Vicovaro et al., 2020; Vicovaro & Burigana, 2014, 2016; White, 2009), supporting the hypothesis that social causality and animacy perception are deeply rooted in early visual processing (Scholl & Gao, 2013), being largely automatic and resistant to higher level beliefs and intentions.

We also see a similarity between this effect and the speed overestimation of the launched object previously found in mechanical causality (Parovel & Casco, 2006). In that case, the authors attributed the speed overestimation to the transmission of an amount of speed from the first moving object, namely, the launcher, to the second object. A similar result has been recently reported by Vicovaro et al. (2020), who proposed that the impetus transmission heuristic can also be conceived as a specific case of a general heuristic about cause–effect relationships, by which a greater cause implies a greater effect. Following this perspective, not only mechanical properties but also psychological properties could be transmitted from one object to another (see White, 2009). In our stimuli, from a phenomenal point of view, as suggested by the free reports collected and analysed in Parovel et al. (2018), the escaping object looks fast but also afraid, as if an internal-psychological factor would influence its motion, while the caterpillar chasing object looks instead frightening. In other words, we suggest that the apparent kinematics of the event could be emphasized by the underlying emotional causes of that motion. As has been well demonstrated by the *ksd principle* (Runeson & Frykholm, 1983; Runeson et al., 2000), perceptual kinematics is mostly directly perceived not for itself but in terms of its causal dynamics, that is, the physical or social forces underlying a perceptual phenomenon.

A possible explanation for this effect could also be found in the ecological valence of adaptive motor skills and behaviours in events such as chasing and escaping. These stimuli, whether they directly challenge the observer or show an interaction between two external agents as in our displays, generally require rapid adaptive behavioural responses, such as evading a threat or escaping from a common danger like a predator; so it should be a strong advantage for an organism if the perceptual processing of these stimuli would be prioritized and the sensitivity to these events, as they were alarm signals, increased (see Brosch et al., 2010). We could even go so far as to hypothesize that the speed of the event in its entirety, namely, of both the chaser and the chased, can be overestimated. Further studies could explore this possibility. Other studies adopting displays containing simple moving geometrical shapes, with one shape pursuing another (i.e., the *wolf* and the *sheep*), already showed that the human visual system is extremely sensitive in detecting chasing (Dittrich & Lea, 1994; Gao et al. 2009). The ability to discriminate between a chasing situation and an emotionally neutral motion, similarly to the ability to discriminate between animate motion and not animate event, would often have direct implications for fitness and survival (see Scholl & Gao, 2013), suggesting that the purpose of vision is not only to recover the physical structure of the local environment but also to recover its causal and social structure (Runeson & Frykholm, 1983; Scholl & Gao, 2013), to build up adaptive behaviours and enhance the learning of social skills, such as quick predator escaping, but even emotional understanding and empathy. We hope that the novel results reported in this article will stimulate research topics focusing on the relationship between qualitative and quantitative properties of motion to enlighten the still enigmatic nature of the relationship between perception and emotion.

## Note

1 Consistency of judgements is assessed by testing WST, Moderate Stochastic Transitivity (MST), and Strong Stochastic Transitivity (SST). If Pxy, the probability of choosing stimulus x over stimulus y, and Pyz, the probability of choosing stimulus y over stimulus z are both ≥0.5, then WST implies that Pxz≥0.5, the MST implies that Pxz≥min{Pxy, Pyz}, and SST implies that Pxz≥max{Pxy, Pyz}. Violations of the WST indicate that it is impossible to derive an ordering of the stimuli, while violations of the other two types of transitivities have different severity. The EBA model requires only that there are no systematic (vs. at random) violations of the MST (but tolerates the presence of systematic violations of SST that sometimes are found when there are subgroups of similar stimuli), while the BTL model assumes that also SST holds and, therefore, that there are no systematic violations of this type of transitivity. A significant goodness-of-fit test for a BTL model is evidence for a systematic violation of the SST, while a significant goodness-of-fit test for an EBA model is evidence for a systematic violation of the MST. For further details, please see Choisel and Wickelmaier (2007); Tversky (1969).
